# Acute Effects of Caffeine Ingestion on Postural Balance, Functional Capacity and Cognitive Function in Patients with End Stage Renal Disease

**DOI:** 10.3390/brainsci14070701

**Published:** 2024-07-13

**Authors:** Hayfa Ben Haj Hassen, Achraf Ammar, Amal Machfer, Nadia Fkih, Siwar Erriahi, Sirine Hamdi, Hamdi Chtourou, Mohamed Amine Bouzid

**Affiliations:** 1 Research Laboratory Education, Motricity, Sport and Health, EM2S, LR19JS01, University of Sfax, Sfax 3000, Tunisia; hayfabenhajhassen@hotmail.com (H.B.H.H.); amalmachfer@gmail.com (A.M.); fekihnadia6@gmail.com (N.F.); siwarerriahi@gmail.com (S.E.); sirinehamdi487@gmail.com (S.H.); bouzid.mohamed-amine@hotmail.fr (M.A.B.); 2 Department of Training and Movement Science, Institute of Sport Science, Johannes Gutenberg-University Mainz, 55099 Mainz, Germany; 3 Research Laboratory, Molecular Bases of Human Pathology, LR19ES13, Faculty of Medicine of Sfax, University of Sfax, Sfax 3029, Tunisia; 4 Interdisciplinary Laboratory in Neurosciences, Physiology and Psychology: Physical Activity, Health and Learning (LINP2), UFR STAPS (Faculty of Sport Sciences), Paris Nanterre University, 92000 Nanterre, France; 5 High Institute of Sport and Physical Education of Sfax, University of Sfax, Sfax 3000, Tunisia; h_chtourou@yahoo.fr; 6 Physical Activity, Sport and Health Research Unit (UR18JS01), National Observatory of Sports, Tunis 1003, Tunisia

**Keywords:** end-stage renal disease, postural balance, function cognitive, caffeine, capacity functional

## Abstract

Patients with end stage renal disease (ESRD) display many cognitive and physiological alterations resulting from renal failure and physical inactivity. Caffeine intake has been reported to improve cognitive/physical performance in several studies. However, whether the benefits of caffeine intake apply to patients with ESRD remains unknown. The aim of the present study was to explore the effect of caffeine intake on postural balance, cognitive function and functional capacity in patients with ESRD. In a randomized order, 12 patients with ESRD (age: 33.85 ± 8.57 years; Female: 45.5%) performed a battery of tests after either caffeine (CF) (400 mg) or placebo (PLA) ingestion. Postural balance was evaluated using a stabilometric platform. Functional capacity was assessed with the sit-to-stand and up-and-go tests, and for cognitive performances, reaction time test and a vigilance test were used. Results showed a significant improvement in up-and-go test (*p* = 0.01) and sit-to-stand test performances with CF compared to PLA (*p* < 0.01). Time of reaction test and vigilance test (*p* < 0.01) performances were significantly higher with CF. No significant difference was observed in postural balance between CF and PLA. The results of this study suggest that acute caffeine ingestion positively enhances cognitive function and functional capacity in patients with ESRD.

## 1. Introduction

To stay alive, each year, over 100,000 people with end-stage renal disease (ESRD) in the United States initiate renal treatment therapy, most commonly hemodialysis (HD). Although HD is a life-saving treatment, it has adverse effects on the heart, brain and limbs [1]. Compared to age-matched individuals in the general population, ESRD patients exhibit significantly greater frailty [2]. Frailty in the ESRD population is expected to become a significant concern in clinical practice, as patients with ESRD are living longer due to improved access to and quality of treatment [3]. Numerous studies have linked ESRD-specific factors, such as a less active lifestyle and lower physical performance, to the higher rate of frailty. However, frailty may also be influenced by a wide range of other chronic kidney disease (CKD)-related factors, such as comorbid conditions, uremic neuropathy, osteopathy, weakness, inflammatory status, reduced cardiovascular function and cognitive function [4,5].

The elevated incidence of falls in the ESRD population, similar to that in frail elderly individuals, is unsurprising given that falls are significant indicators associated with increased frailty levels [6,7] compared to the general population. Recent studies indicate that patients with ESRD exhibit numerous fall risk factors, including gait disorders [8], balance impairments [9] and cognitive decline [10], even in the early stages of renal disease. Considering all these factors, the health status of ESRD patients has emerged as a significant global public health issue.

To preserve and enhance physical and cognitive skills, as well as postural balance, effective interventions are needed for this population. Nutritional aids like caffeine could potentially help address issues with cognitive, functional and postural balance in individuals with ESRD. Caffeine is believed to act as a central stimulant, improving cognitive and psychomotor functions by enhancing reaction time, alertness, vigilance and memory in both older adults and young individuals [11,12]. In terms of functional capacity, studies suggest that acute caffeine intake can enhance power, muscle strength and endurance in various populations, including older adults [13], middle-aged women [14] and the elderly [15].

However, the impact of caffeine on postural stability remains contentious, especially given the existence of various influencing factors such as dosage, experimental procedures, participant characteristics and the studied population. Some research indicates potential improvements [16], decreases [17], or minimal effects [18,19] on standing balance performance, with caffeine dosages ranging from 160 to ~400 mg. Methodological differences and variations in balance tasks and outcome measures further contribute to the inconsistency in findings. Additional research, with more comprehensive assessments, is necessary to better understand the effects of caffeine on balance performance.

Despite previous reports of functional, cognitive and postural balance issues in patients with ESRD and the known positive impact of caffeine in alleviating these issues in both young and older adults, there is a lack of comprehensive data on the immediate effects of caffeine on physical, cognitive and postural balance performances specifically in ESRD patients. A study by Nikić et al. [20] represents a pioneering effort in investigating the beneficial effects of caffeine intake in HD patients. However, this study only focused on cognitive function. Specifically, moderate caffeine intake through habitual coffee consumption was shown to positively affect patients’ cognitive functions, possibly due to selective enhancement of vigilance and attention.

Given the potential significance of identifying low-cost and easily accessible interventions to delay multiple disease-related problems such as postural impairment and cognitive decline in this population, as well as the importance of maintaining daily abilities to support independence, it may be necessary to simultaneously evaluate the potential effects of such interventions on both physical and cognitive abilities. In particular, evaluating the impact of caffeine supplementation on the ESRD population could provide valuable insights.

Therefore, this study aimed to investigate the acute effects of caffeine intake on functional, cognitive and postural balance in ESRD patients. We hypothesized that caffeine intake would improve functional and cognitive performances, as well as postural balance, in these patients.

## 2. Materials and Methods

### 2.1. Participants

The minimum required sample size was calculated using the software G*power (version 3.1.9.6; Kiel University, Kiel, Germany). The *t* test family for paired sample “Means: Difference” between two dependent means (matched pairs) and the “A priori” type of power analysis were selected. The probability of type I error (α) was fixed at 0.05. Given the difficulty in accessing a large sample size of patients with ESRD, the power (1-β error probability) was fixed at 0.8, the minimal required power value based on a balance between feasibility and reliability. This threshold value was recommended by Cohen [21] as a reasonable balance in many research contexts where recruiting a large sample size can be impractical in terms of time, cost and participant availability.

Based on the power calculation procedure by Filip-Stachnik et al. [22], employing an effect size of 0.89 and discussions among the authors, the effect size was set at 0.8 for the present study. The analysis revealed that a minimum of 12 participants would be sufficient to reduce the likelihood of committing a type II statistical error, ensuring an actual power of 82.9%.

Twelve patients with ESRD (age: 33.85 ± 8.57 years; female: 45.5%, weigh: 70.91 ± 19.27 kg, height: 167.18 ± 12.28 cm, body mass index: 25.48 ± 7.18 kg m^2^; mean ± SD), volunteered to participate in this study (Table 1). They were recruited through the Ksour Essef Dialysis Unit and Renal Unit of Taher Sfar Hospital in Mahdia, Tunisia. All subjects gave informed consent for study participation. The study received approval from the Regional Research Ethics Committee (CPP SUD N° 0459/2022) and was registered with the Pan-African Clinical Trial Registry (PACTR202206634181851). The study followed the ethical principles of the Declaration of Helsinki. Table 1 presents the detailed characteristics of the patients.

### 2.2. Inclusion and Exclusion Criteria

Eligible participants were male or female, over 18 years of age, diagnosed with ESRD (stage 5 chronic kidney disease; glomerular filtration rate < 15 mL.min^−1^. 1.73 m^−2^) and had undergone hemodialisys for a minimum of 12 weeks. Participants were excluded if they engaged in regular physical activity or sport (>150 min·wk^−1^) or in excessive sport activities; if they had persistent uncontrolled blood pressure, clinically significant or symptomatic cardiovascular or peripheral vascular disease, or any musculoskeletal limitations or neurological conditions; if they were current smokers; or if they had required hospitalisation for non-dialysis reasons in the 4 weeks prior to the study.

### 2.3. Study Design and Supplementation Protocol

This is a randomized, controlled, double-blind trial. Participants were instructed to avoid strenuous exercise and maintain their normal dietary patterns 48 h prior to testing. Additionally, they were instructed not to consume caffeine after 6:00 P.M. the night before testing to account for any caffeine already ingested. A 12-h fasting period was deemed sufficient for caffeine clearance, considering its plasma half-life and elimination rate of 2.5–5 h in healthy individuals [23]. Participants made three visits to the laboratory, each separated by at least a 4-day interval. During the initial visit, they were familiarized with the study procedures. Notably, in this familiarization session, no capsules (either containing a placebo or caffeine) were administered.

In the subsequent two experimental sessions, participants underwent cognitive, functional and postural balance tests 60 min after consuming either a 400 mg caffeine capsule or a placebo, following a randomized order. This timing was chosen because plasma caffeine levels peak around this time [24]. The caffeine capsules (CF) contained 400 mg of caffeine, while the placebo (PLA) contained no caffeine. According to Grgic et al. [25], the majority of studies investigating the effects of caffeine ingestion used a single dose of around 6 mg/kg, equivalent to approximately 400 mg, which is considered a higher dose without notable side effects. Furthermore, Jenkins et al. [26] demonstrated that consuming 3–9 mg/kg (approximately 200 to 600 mg) of caffeine induced similar benefits.

All treatment capsules in the present study (CF or PLA) were prepared by a chemist. For each test session, each participant consumed an assigned treatment capsule (CF or PLA) with a cup of water following the double-blind method. All test sessions were performed at the same time of day (specify the time) to avoid the potential impact of diurnal variation [27,28]. Additionally, all participants were requested to maintain their normal sleep patterns the night preceding each test session, with a recommended sleep duration of 7–9 h.

### 2.4. Assessments of Cognitive Function

#### 2.4.1. Vigilance Test (VT)

The vigilance task (VT) involved identifying a specific symbol (a figure consisting of three numbers) and circling it as quickly as possible within a set time limit of one minute. Participants were required to work line by line from left to right, ignoring all other figures that were not composed of three numbers. The test sheet contained a total of 600 symbols, organized into 36 lines. Performance on the test was evaluated based on the total number of symbols circled by each participant, reflecting their vigilance performance [29].

#### 2.4.2. Reaction Time Test (RTT)

The reaction time to a visual stimulus was assessed using the INRP 2005–2009 program (F. Jauzien, version 4.05). The reliability of this test was previously reported [30]. Participants were seated at a distance of 0.4 to 0.5 m in front of a computer screen. Following ten familiarization trials, each participant performed 20 simple reaction time tests in a randomized, double-blind manner. The stimulus consisted of a blue square displayed for 50 milliseconds at the center of the screen, preceded by a white square stimulus to direct their fixation. After a training trial, participants were instructed to press a specific computer key as quickly as possible using their preferred hand upon the appearance of the blue square. The response time score was determined by calculating the mean reaction time across the 20 trials. Reaction times falling below 150 milliseconds or exceeding 800 milliseconds were excluded from the analysis to avoid the influence of anticipation or momentary lapses in concentration

### 2.5. Assessments of Postural Balance

Participants’ standing postural balance was evaluated using a static stabilometric platform (PostureWin©, Techno Concept^®^, Cereste, France; sampling frequency of 14 Hz, 12-bit A/D conversion) that recorded centre of pressure (COP) displacements [31]. They were instructed to maintain an upright posture as still as possible, adopting a comfortable and natural posture with eyes open and fixed on a cross positioned approximately 3 m away at eye level. Participants stood barefoot with feet shoulder-width apart on the platform, arms relaxed by their sides and head facing forward. Each participant maintained a quiet stance for 25.6 s, following the guidelines of the French Posturology Association. Four COP sway parameters were analysed to assess participants’ postural balance: COP area, COP length (sum of COP displacement in the medial-lateral direction, COPx and antero-posterior direction, COPy) and mean COP velocity (COP_Vm_).

### 2.6. Assessments of Functional Capacity

#### 2.6.1. Time Up-and-Go Test (TUGT)

Lower-body strength and endurance were assessed using the 30-s chair stand test (CS-30). Participants were instructed to sit in a chair of standard height with their arms crossed over their chest. They were then asked to rise to a standing position and return to a seated position as many times as possible within a period of 30 s.

#### 2.6.2. Sit-to-Stand (STS30)

Functional mobility was evaluated using the timed up-and-go test (TUG). Participants were timed as they rose from a straight-backed chair with a height of 45 cm, walked a distance of 3 m, turned around and then returned to their initial sitting position.

### 2.7. Statistical Analysis

Descriptive statistics (mean ± SD) were calculated for all variables. The Shapiro–Wilk test was conducted to confirm the normality of data distribution. The functional, cognitive and postural performances were analysed using paired Student’s *t*-test. Additionally, effect size was calculated using Cohen’s d with the following classifications: small (≦0.49), medium (0.50–0.79), large (0.80–1.19), very large (1.20–1.99) and huge (≧2.00) [32]. STATISTICA software version 12.0 (StatSoft, France) was used for all analyses, and statistical significance was set at *p* < 0.05.

## 3. Results

### 3.1. Functional Capacity

**Figure 1:**
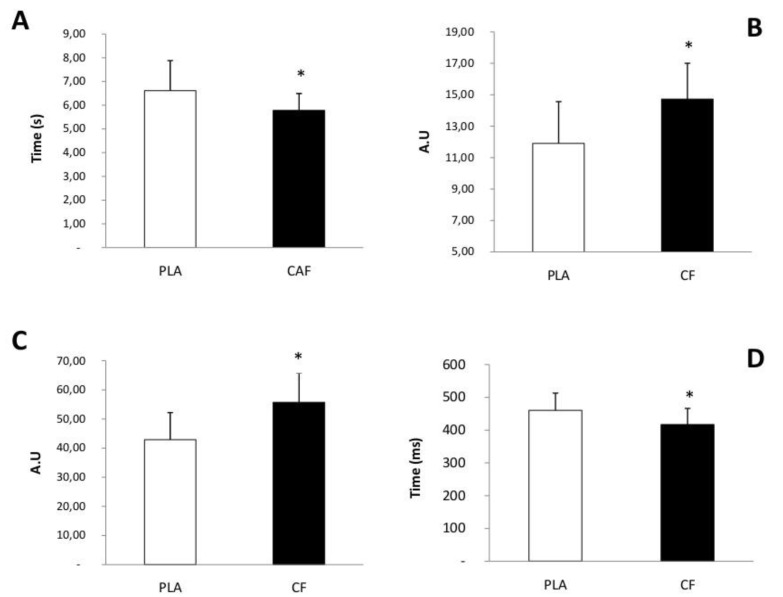
Summary for up-and-go test (**A**), sit-to-stand test (**B**), vigilance test (**C**) and reaction time test (**D**) performances in the caffeine and placebo intake conditions (mean ± SE). *: significant difference to placebo condition.

Statistical analysis showed that, compared to PLA condition, time in the up-and-go test was significantly shorter in the CF sessions (t = 3.06, *p* = 0.01, d = 0.48) (Figure 1A). Additionally, performance in sit-to-stand test was significantly higher in CF compared to PLA session (t = 7.038, *p* < 0.01, d = 0.83) (Figure 1B).

### 3.2. Cognitive Function

Statistical analysis showed that the number of correct answers in the vigilance test was significantly higher under CF conditions (t = −4.99, *p* < 0.01, d = 0.96) (Figure 1C). Moreover, the time in reaction time test was significantly shorter under CF compared to PLA condition (t = 3.92, *p* < 0.01, d = 0.60) (Figure 1D).

### 3.3. Balance Postural Performance

During the posturographic recording, our results did not reveal any significant difference in the COParea (t = 0.05, *p* = 0.60, d = 0.02), COPx (t = 1.64, *p* = 0.13, d = 0.21), COPy (t = −0.85, *p* = 0.41, d = 0.06) and COP_vm_ (t = 1.30, *p* = 0.22 d = 0.14) between the two conditions (Table 2).

## 4. Discussion

The aim of our study was to evaluate the acute effects of CF ingestion on postural balance, cognitive function and functional capacity in patients with ESRD. The main results showed that, compared to PLA, CF ingestion enhanced cognitive and functional capacity but with no significant effects on postural balance.

Research has shown that increased caffeine intake is linked to a reduced risk of developing chronic kidney disease (CKD), with each additional cup consumed correlating to a 3% decrease in CKD risk [33]. This association is particularly significant for adults with metabolic syndrome [34]. Furthermore, higher caffeine consumption has been associated with lower overall mortality rates among patients diagnosed with CKD [35].

In the present study, the significant improvement in cognitive performance with CF ingestion in ESRD subjects align with previous research indicating that caffeine consumption improves cognitive function in healthy young people [36], aged women [11], older adults [37] and hemodialysis patients [20]. However, other studies have reported no effects of high caffeine consumption (≥400 mg) in cognitive performance [38,39]. In addition, previous studies have reported that high caffeine consumption can have harmful effects including anxiety, loss of balance, bad mood and inattention [40,41]. This discrepancy between the studies’ results could be explained by a difference in protocols of caffeine administration including time of ingestion, mode of caffeine (pure, coffee, drink) and dose (low, moderate, high) [14].

The mechanisms by which caffeine improves cognition in patients with ESRD in the present study steel unclear. However, a possible explanation could be that CF ingestion binds to adenosine receptors (A1 and A2A) and blocks the action of agonists on these receptors that are closely associated with alertness regulation [42]. Moreover, given that adenosine has been associated with an inhibition of the release of excitatory neurotransmitters, such as dopamine, CF indirectly increases the available amount of dopamine in the brain [43] which could increase attention [44], improves wakefulness and therefore cognitive performance [45].

Regarding the effects of CF ingestion on functional capacity, the current data showed that consumption of caffeine improved functional performance, including muscle endurance and functional mobility in ESRD subjects. Our findings are in agreement with those reported by Waer et al. [14] and Duncan et al. [11]. A recent review by Grgic et al. [46] noted that caffeine consumption improves physical performance, including maximum strength and power and muscular endurance, in sedentary and active subjects. It is important to note that this beneficial effect of caffeine is not general and depends on the dose ingested. Indeed, recent studies have shown that low-dose consumption of caffeine did not result in improved functional performance [14,47]. From a mechanistic perspective, the observed enhancement in functional capacity in our study could be attributed to the impact of caffeine on the central nervous system through adenosine inhibition [48]. Particularly, adenosine increases in the brain during stress and in plasma and muscles during muscle activity, negatively influencing neuron excitability and synapse transmission. Therefore, antagonism of adenosine due to caffeine ingestion would improve physical performance in ESRD participants.

Concerning postural balance, our results showed no significant difference in static bipodal postural balance between PLA and CF ingestion in ESRD subjects. These findings are in line with the studies of Enriquez et al. [18] and Norager et al. [49]. However, Ben Waer et al. [50] demonstrated that caffeine consumption improves postural balance of middle-aged women under specific conditions. In fact, in this study, the authors reported that COP_Vm_ values decreased significantly after CF ingestion, but only when visual and somatosensory inputs were perturbed. These results suggest that the effect of CF on postural balance does not depend on the manipulation of sensory functions. In the same way, Kim et al. [51] demonstrated that caffeine consumption seems to improve postural balance in stroke patients, but only after visual deprivation. Similarly, Kara et al. [16] demonstrated that caffeine was associated with improved standing balance performance during specific tasks in young adults. These authors proved that the effects of caffeine were observed in relation to specific combinations of visual input (eyes opened vs. eyes closed) and perceptual–cognitive tasks. In the present study, the absence of CF ingestion effects on postural balance could be related to the postural experimental condition. In fact, postural balance was evaluated with opened eyes and a firm surface. Such a simple postural experimental condition would not affect mechanoreceptors and visual input of postural balance in ESRD patients. Therefore, CF ingestion effects on postural balance would not be detectable in a simple condition. Further studies using different postural conditions (foam surface/Eyes closed) would help to comprehensively verify CF ingestion effects in patients with ESRD.

### Strength and Limitation

This study represents a pioneering effort to assess the acute effects of CF ingestion on both physical and cognitive performance among ESRD patients. Nevertheless, the study’s design precludes generalization and it is crucial to acknowledge several limitations that should be considered when interpreting the present findings. First, although the study’s sample size could achieve a statistical power of 0.8, meeting recommended values, the number of participants is considered low due to recruitment challenges among ESRD patients. The study protocol, which involves several tests, was declined by many potential participants. Second, the habitual caffeine consumption of participants was not controlled, and we used only one dose of caffeine (400 mg) in the present study. Future studies may consider examining other dose-response relationships to caffeine ingestion in patients with ESRD, taking into account different habitual consumption levels. Lastly, incorporating additional types of sensory perturbation (such as vestibular inputs) and/or concurrent tasks could help provide a clearer explanation of the postural balance results.

## 5. Conclusions

The results of the present study indicate that acute caffeine ingestion positively enhances functional and cognitive performance in patients with ESRD, but without significant effects on postural balance. This improvement could offer greater potential for everyday functioning and dependence, which could improve the quality of life in this population. However, it is important to note that the sample size in this study was relatively small (n = 12). To strengthen the validity and generalizability of these findings, future studies with larger sample sizes are recommended. These studies should also consider genetic and geographical variables to provide a more comprehensive understanding of the effects of caffeine ingestion in this population.

## 6. Practical Implications

These findings suggest that consuming 400 mg of caffeine, equivalent to approximately two cups of instant coffee, could potentially enhance motor and cognitive performance in ESRD patients. This improvement could be particularly beneficial for everyday activities that require controlled balance (such as walking and standing) while simultaneously performing tasks (e.g., when crossing a busy intersection: talking, scanning the busy street for threats, or tracking visual targets).

## Figures and Tables

**Table 1:** Participant characteristics (mean ± standard deviation).

Total (n)	12
Sex. *n* (%)	
Female	5
Male	7
Age (years)	34 ± 8.98
Weight (kg)	70.91 ± 19.27
Height (cm)	167.18 ± 12.28
BMI (kg/m^2^)	25.48 ± 7.18
Hours in bed	7.69 ± 1.18
**Comorbidities**	
Hypertension (%)	3 (24%)
Glycemic (%)	1 (8%)
Clinical parameters	
Time in dialysis (months)	36.12 ± 11.96
eGFR (mL/min/1.73 m^2^)	7.95 ± 2.96
Hb (mg/dL)	111.01 ± 10.14
**Usual daily nutrient intake**	
Total caloric (TC) intake (kcal)	1920.0 ± 277
Protein (% of TC)	16.2 ± 2.3
Fat (% of TC)	37.4 ± 4.1
Carbohydrate (% of TC)	46.4 ± 6.5

BMI: body mass index, eGFR: estimated glomerular filtration rate, Hb: hemoglobin.

**Table 2:** Summary for postural performance in the caffeine and placebo intake condition (mean ± SE).

	Placebo	Caffeine	*p*	ES
Postural performance				
COP_Vm_ (mm/s)	7.77 ± 1.95	8.79 ± 3.08	0.22	0.14
COParea (mm^2^)	178.56 ± 78.28	153.45 ± 86.27	0.60	0.02
COPx (mm)	214.60 ± 65.80	275.11 ± 92.43	0.13	0.21
COPy (mm)	279.48 ± 87.70	317.83 ± 100.73	0.41	0.06

COParea: center of pressure area, COPx: oscillating of COP in the sagittal plane in the COPy: oscillating of COP in frontal plane, COP_Vm_: mean center of pressure velocity, ms: milliseconds, s: second, mm: millimeter, mm/s: millimeters per second, mm^2^: square millimeter.

## Data Availability

The raw data supporting the conclusions of this article will be made available by the authors on request. The data is not publicly available due to privacy concerns.
